# Management of late biliary complications in pediatric liver transplant recipients

**DOI:** 10.3389/fgstr.2025.1653955

**Published:** 2026-02-18

**Authors:** Davide Cussa, Michele Pinon, Andrea Doriguzzi Breatta, Marco Fronda, Pier Luigi Calvo, Renato Romagnoli

**Affiliations:** 1 Dipartimento di Chirurgia Generale e Specialistica, Azienda Ospedaliero Universitaria Città della Salute e della Scienza di Torino, Turin, Italy; 2 Dipartimento Diagnostica Per Immagini E Radiologia Interventistica-Aou Citta Della Salute Di Torino, Torino, Italy; 3 Universita degli Studi di Torino Dipartimento di Scienze della Sanita Pubblica e Pediatriche, Turin, Italy

**Keywords:** biliary com, interventional radiology, liver transplant, pediatric, transplant surgery

## Abstract

The number of pediatric liver transplant recipients with long-term follow-up exceeding 20 years is steadily increasing. These patients are characterized not only by their extensive medical histories but also by their long future life expectancy. In this context, careful management of post-transplant complications, including biliary issues, is essential. We identified 40 patients from our 193 pediatric transplants performed since the program’s inception in 1995, with more than 20 years of follow-up at our center. Thirteen of these patients developed either early or late biliary complications. Five developed complications within the first post-transplant year, while eight developed late complications, which are the main focus of this study. We detail the management of biliary complications in these patients, providing an in-depth analysis of four case models and an overview of the remaining patients. In addition to the standard interventional options, such as percutaneous bilioplasties and surgical revisions of anastomoses, we identified a subgroup that may benefit from a more conservative approach, provided they are closely monitored through a rigorous follow-up protocol.

## Introduction

Biliary complications are among the most significant issues following liver transplantation, affecting approximately 20-30% of patients. Pediatric liver transplant recipients are no exception, and pediatric transplantation itself may pose a risk for the development of biliary complications. This is particularly true in cases where reduced grafts are used, such as left-sided splits or livers from living donors, which often involve extremely delicate bile ducts and require complex reconstructions, including bilio-digestive anastomoses sometimes double-duct—or biliary plasty with enlargement (1, 2).

Early biliary complications (occurring within the first 6 months to 1 year post-transplant) are most commonly represented by fistulas or anastomotic strictures. Additionally, bile leaks from reduced graft segments are frequent and typically have significant clinical impact, often requiring interventional approaches. These range from endoscopic therapies (ERCP) to interventional radiology procedures with placement of percutaneous biliary drains or possible bilioplasties (3, 4). In more complex or refractory cases, relaparotomy and redo anastomoses may be necessary (5).

The management of late-onset biliary complications, however, presents more complex decision-making challenges. These complications include delayed anastomotic strictures, particularly in cases involving bilio digestive anastomoses, or ischemic bile duct lesions (ITBL). The incidence of ITBL is reportedly increasing due to the systematic use of extended criteria donors, such as DCD (donation after circulatory death) or elderly donors (6). Nonetheless, it is believed that the use of perfusion machines, which have proven to be safe and effective even for pediatric liver transplants, may partially mitigate their impact (7–9).

Late biliary complications can severely affect the quality of life in pediatric transplant recipients, manifesting with symptoms such as jaundice or pruritus, which are often refractory to treatment, or severe and recurrent cholangitis. As a result, repeated invasive treatments are typically the first-line approach in transplant centers (8). Clinicians are also aware that repeated acute episodes of biliary damage can worsen the overall clinical picture, affecting both the biliary tree and liver function, ultimately leading to re- transplantation in 2-5% of patients or, in rare and complex cases, mortality rates of 1-2% (5).

An increasingly prominent group of patients in the international transplant landscape comprises those who underwent transplantation during childhood and have now reached 20–30 years of follow-up. These individuals still have a long life expectancy ahead, but there is little to no data available on long-term graft survival and quality of life beyond 20 years (with only a few reports in the literature extending beyond this period) (9). In these patients, managing late biliary complications becomes particularly challenging, as the long history of the graft and the need to ensure good quality of life for several more years must be taken into account (9, 10).

In this context, the available literature is extremely scarce, and the management of biliary complications requires a tailored approach, with strategies defined on a case-by-case basis. Our experience as a center with 34 years of activity and more than 4,000 liver transplants performed, including approximately 200 pediatric transplants, is situated within this highly specific patient setting (5).

## Patients and methods

Since 1995, the year the pediatric transplant program was launched at our center, 192 children have undergone liver transplantation. The follow-up of these patients has essentially been “lifelong,” although the frequency and types of examinations have gradually decreased over time.

Initially, a very close follow-up was conducted, with blood tests every 15 days after discharge from the inpatient unit, accompanied by an ultrasound examination. Over time, the frequency of follow-up decreased to approximately every six months, involving ultrasound, clinical examination, and blood tests, and became annual after 10 years post-transplant.

At our center, we still perform protocol biopsies at 1, 3, and 5 years post-transplant, and then every five years for subsequent follow-up. Additional second-level tests, such as CT scans or MR cholangiography, are ordered only if clinically indicated, as is the case for endoscopic examinations.

In this cohort of patients, we identified those with a minimum of 20 years of follow-up to investigate the presence of long-term biliary complications, excluding patients who had experienced early biliary complications within the first year post-transplant.

For all patients with more than 20 years of follow-up, we reviewed their medical records, reports from outpatient follow-up visits, trends in blood test results, and immunosuppressive therapies.

For patients in whom we found documented biliary complications, either clinically or through second-level tests such as MR cholangiography, we collected data on hospital admissions, endoscopic or radiological interventional procedures, and the need for surgical re-intervention or re-transplantation.

Additionally, we identified four “model” clinical cases, in which we chose to describe our approach to foster discussion on what could have been done differently and to share the lessons learned from managing these complex cases in such a specific patient population.

In our series, we additionally excluded four patients who underwent retransplantation for biliary complications (2% of cases), as they were retransplanted before reaching 20 years of follow-up and therefore did not meet the study’s inclusion criteria.

## Results

We included 40 patients in this study with a minimum follow-up of 20 years post liver transplantation.

### Characteristics and data of the included patients

The median age at liver transplantation (LT) was 1.4 years, with 29 recipients (72.5%) being male. Biliary atresia was the most common indication for LT, accounting for 60% (24 cases). Nineteen patients (47.5%) received a whole graft from a deceased donor, 16 patients (40%) received a left lateral segment graft from a deceased donor, and 5 patients (12.5%) received a left lateral sector from a living donor. Three patients underwent combined liver-kidney transplantation, with all grafts coming from deceased donors. Ten patients (25%) underwent retransplantation, and 2 patients (5%) were retransplanted twice.

At the time of the last follow-up, the majority of patients (31, 77.5%) were on tacrolimus-based immunosuppression, with 20 on a once-daily regimen and 11 on a twice-daily regimen. Three patients (7.9%) were on cyclosporin A or mycophenolate mofetil monotherapy. Only one patient had discontinued immunosuppressive therapy, choosing to stop the medication voluntarily, which did not result in acute rejection. Since this was discovered months later, immunosuppressive therapy was not resumed.

The median follow-up was 23 years (range: 21–27.8 years). Patient survival rates at 10 and 20 years were 97.5% (95% confidence interval: 92.8–100%), while graft survival rates at 10 and 20 years were 77.5% (65.6–91.6%) and 74.8% (62.5–89.6%), respectively. The only death in this series was due to post-transplant lymphoproliferative disease. Causes of graft loss varied based on timing. Among the four patients who lost their grafts within 90 days of LT, three (75%) experienced hepatic artery thrombosis, and one (25%) had primary non-function. Seven grafts were lost more than 90 days after LT due to chronic rejection (4 patients, 57.1%), *de novo* autoimmune hepatitis (1 patient, 14.3%), death with a functioning graft (1 patient, 14.3%), and an undetermined cause (1 patient, 14.3%). The general characteristics and data of these 40 patients are summarized in **Table 1**.

**Table 1:** Patient characteristics with ≥20 years of follow-up.

Variable	**Number (%)**
Age at transplantation [median (range)]	1.4 (range) years
Male recipients	29 (72.5%)
Whole graft from deceased donor	19 (47.5%)
Left lateral segment from deceased donor	16 (40%)
Left lateral sector from living donor	5 (12.5%)
Combined liver–kidney transplantation	3 (7.5%)
Retransplantation cases	10 (25%)
Twice retransplanted	2 (5%)
Tacrolimus-based immunosuppression	31 (77.5%)
Once-daily tacrolimus regimen	20 (50%)
Patient survival (10 years)	97.5%
Patient survival (20 years)	97.5%
Graft survival (10 years)	77.5%
Graft survival (20 years)	74.8%

### Biliary complications

#### Early biliary complications

With regard to biliary complications 5 patients (12.5%) experienced early biliary complications within the first year post-transplant. These included: 3 anastomotic biliary fistulas, one of which was subclinical and managed by maintaining surgical drainage, while the other two required relaparotomy; a severe stricture of the biliodigestive anastomosis that occurred approximately 6 months post-transplant, treated with bilioplasty; and a significant bile leak from the transection of the left split, involving a duct excluded from the anastomosis, which required surgical reintervention.

#### Late biliary complications

In our analysis of late-onset biliary complications, the focus of this study, we identified 8 patients who developed biliary complications during follow-up, occurring more than one year after transplantation. This brings the total number of patients with biliary complications in this cohort to 13, representing 30% of the patients.

All 8 patients who experienced late biliary complications had undergone biliodigestive anastomosis and developed either anastomotic strictures or intrahepatic bile duct stenosis and dilations, suggesting an ischemic component in the pathogenesis of these issues. All patients investigated for late biliary complications had experienced at least one episode of cholangitis during their follow-up. In 4 cases (50%), biliary complications were clinically evident during episodes of cholangitis, while in the other 4 cases (50%), they were discovered during investigations for elevated GGT levels. In all cases, the diagnosis was confirmed through MR cholangiography.

Four patients (50%) required treatment: 3 underwent percutaneous interventional procedures, while 1 required revision of the biliodigestive anastomosis. Four patients (50%) are currently under close follow-up with elevated GGT levels (all > 400 U/L), either asymptomatic or with mild symptoms, such as pruritus, which is well-controlled with antihistamines. Three patients are currently on prophylactic antibiotic therapy, in agreement with infectious disease specialists.

Among the 40 patients included in this study, no cases of biliary complications led to re-transplantation. However, upon reviewing the data, it is likely that at least one of the patients who underwent re-transplantation for chronic rejection also had significant biliary issues, as indicated by the presence of non- specific “cholangitic episodes” in the medical records, despite the lack of available imaging for this patient.

**Figure 1:**
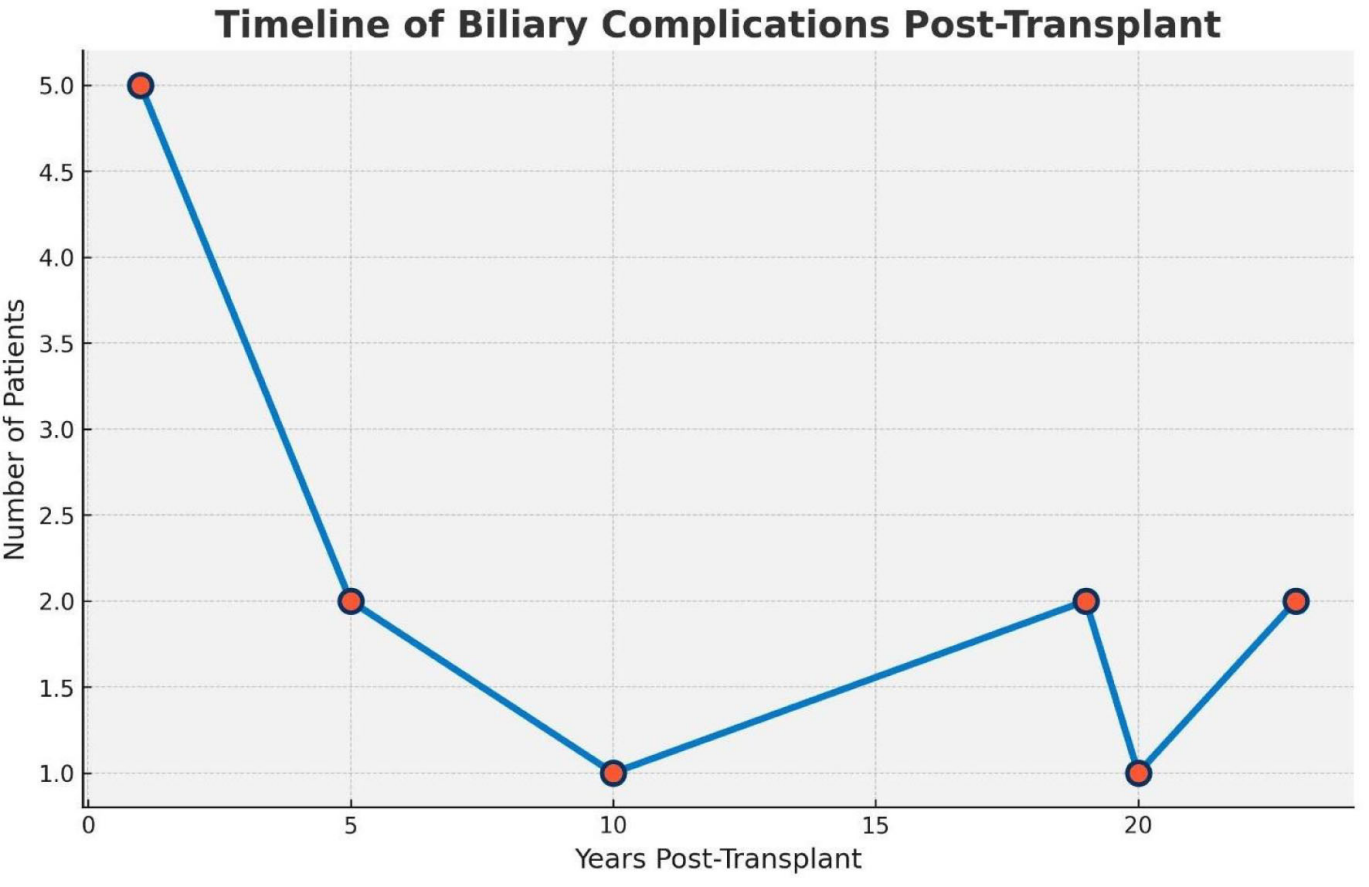
Timing of onset of biliary complications during follow-up. Analysis of patients with late biliary complications.

The characteristics of late biliary complications are presented in **Table 2**, while the timing of complication onset is summarized in the graph in **Figure 1**.

**Table 2:** Clinical characteristics of patients who presented with late biliary complications.

Variable	**Number (%)**
Total patients with late biliary complications	8 (100%)
Bilio-digestive anastomosis with strictures	8 (100%)
Patients with cholangitis episodes	8 (100%)
Complications detected during cholangitis	4 (50%)
Complications detected due to elevated GGT	4 (50%)
Patients requiring treatment	4 (50%)
Percutaneous treatment	3 (37.5%)
Revision of bilio-digestive anastomosis	1 (12.5%)
Follow-up with elevated GGT (>400 U/L)	4 (50%)
Mild symptoms (e.g., pruritus)	4 (50%)
Prophylactic antibiotic therapy	3 (37.5%)
Re-transplant due to biliary complications	0 (0%)
Likely re-transplant due to biliary issues	1 (12.5%)

Percentages are calculated over 8 patients experiencing late biliary complications.

Among the 8 patients identified with late biliary complications, we analyzed 4 cases that we considered ideal for highlighting the complexity of managing these types of complications. These cases also demonstrate how clinical presentation and treatment can vary significantly in this particular setting.

#### Case 1

We analyzed this case as exemplary of the routine management of the most common late-onset complication in this setting, namely, biliodigestive anastomosis stricture.

The case involves a girl who underwent liver transplantation at the age of one for biliary atresia with a left liver split graft. She developed a stricture of the biliodigestive anastomosis 5 years post-transplant, presenting with acute cholangitis, requiring hospitalization and antibiotic therapy. Upon admission, MR cholangiography revealed a stricture of the biliodigestive anastomosis. Blood tests at admission showed a total bilirubin of 6.3 g/dL and GGT of 285 U/L. Given the clinical presentation and the MR cholangiography results, a percutaneous interventional radiology procedure with bilioplasty was performed. Two bilioplasty procedures were required to achieve full clinical and radiological resolution. Subsequent follow-up was uneventful.

**Figure 2:**
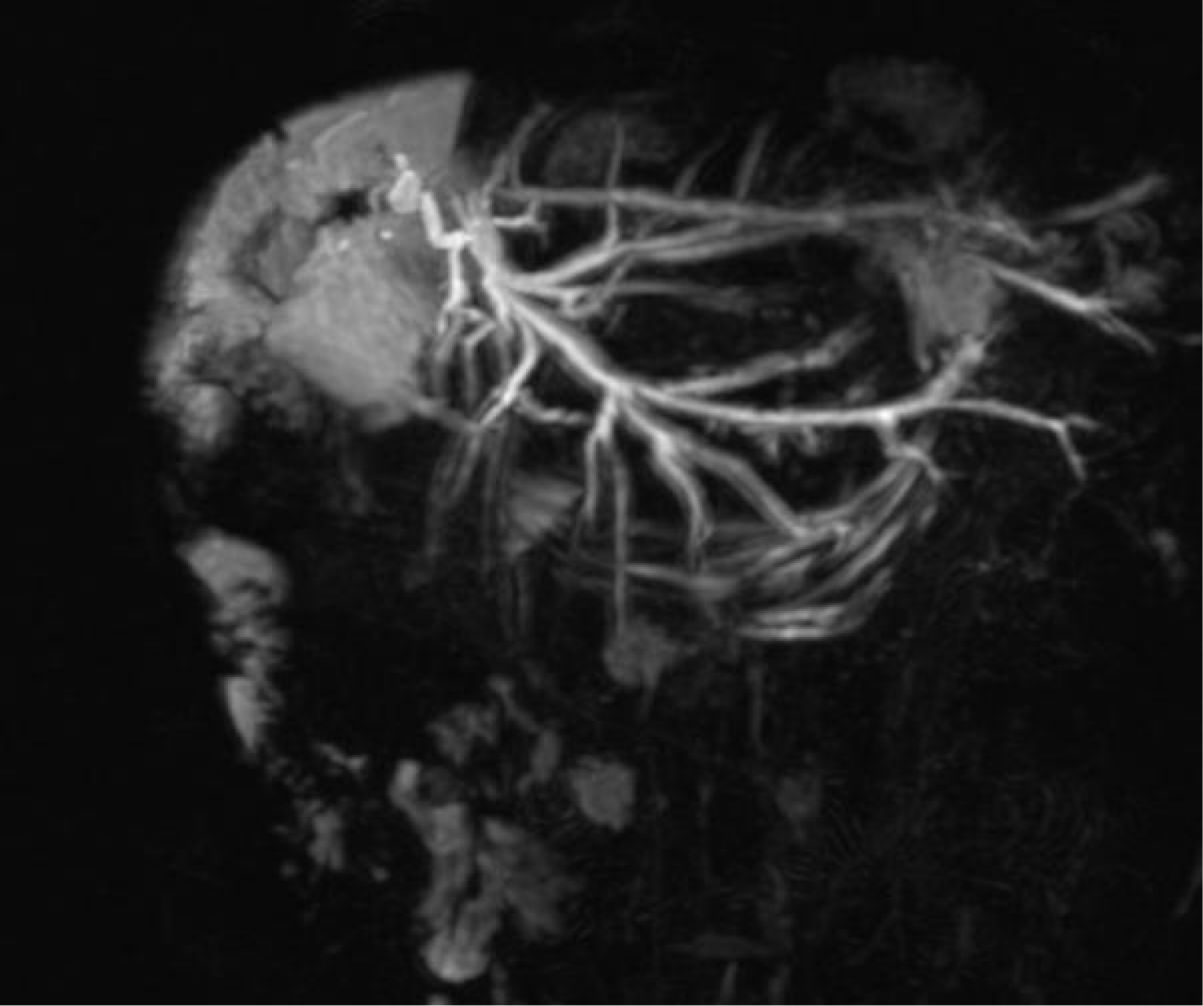
Stricture of the biliodigestive anastomosis following left liver split on MR cholangiography.

**Figure 3:**
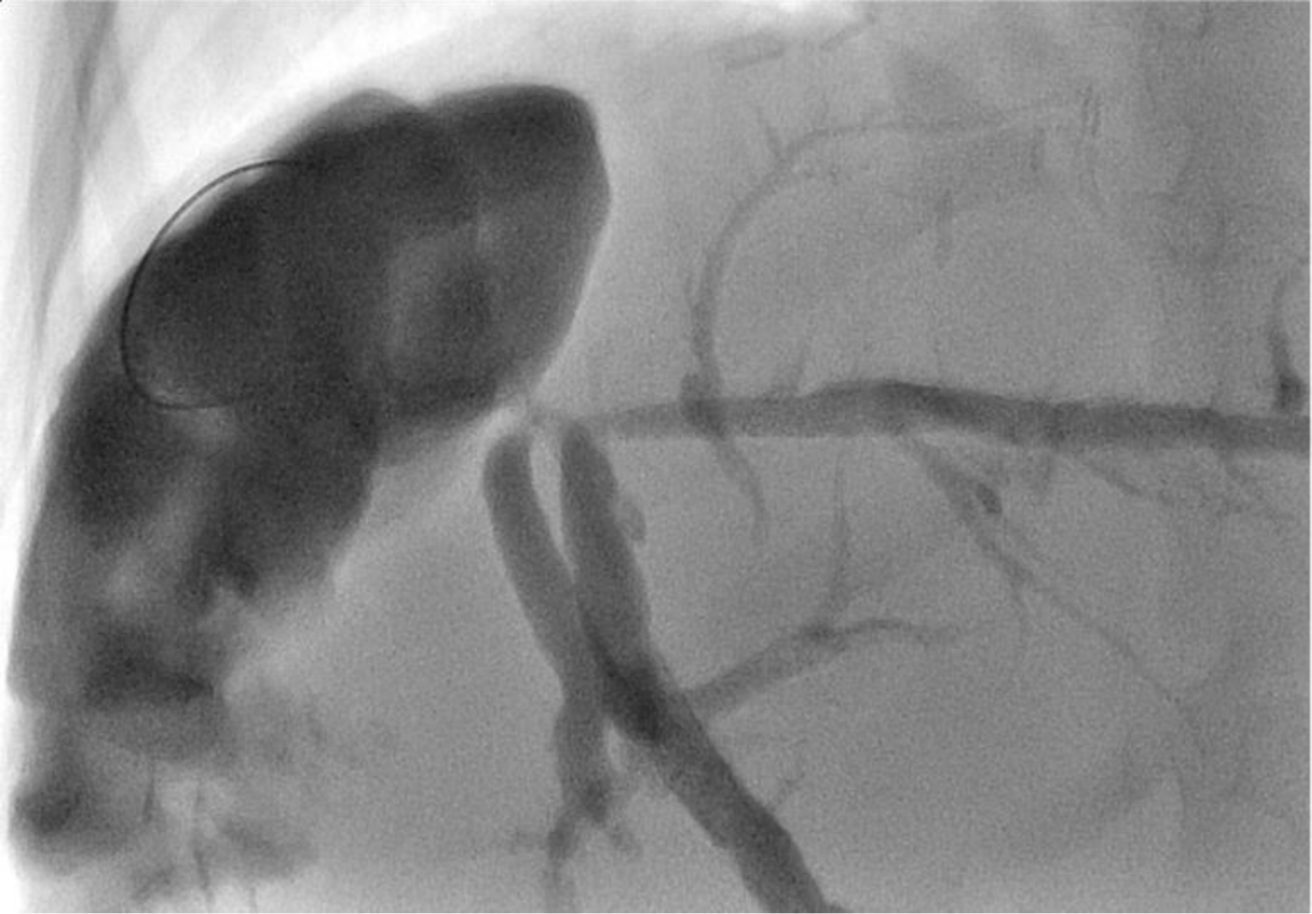
Regular outcome of percutaneous bilioplasty.

The images below show the MR cholangiography (**Figure 2**) and the corresponding cholangiographic images of the bilioplasty (**Figure 3**).

We selected this case as the first example of managing a straightforward and relatively simple case, to use it as a comparison with the more complex cases that follow.

#### Case 2

In the second case we present, we chose to re-evaluate our management approach regarding the biliary complication that arose in a 22-year-old woman who had undergone a liver transplant at the age of 2 for hepatoblastoma, using a left liver split graft.

**Figure 4:**
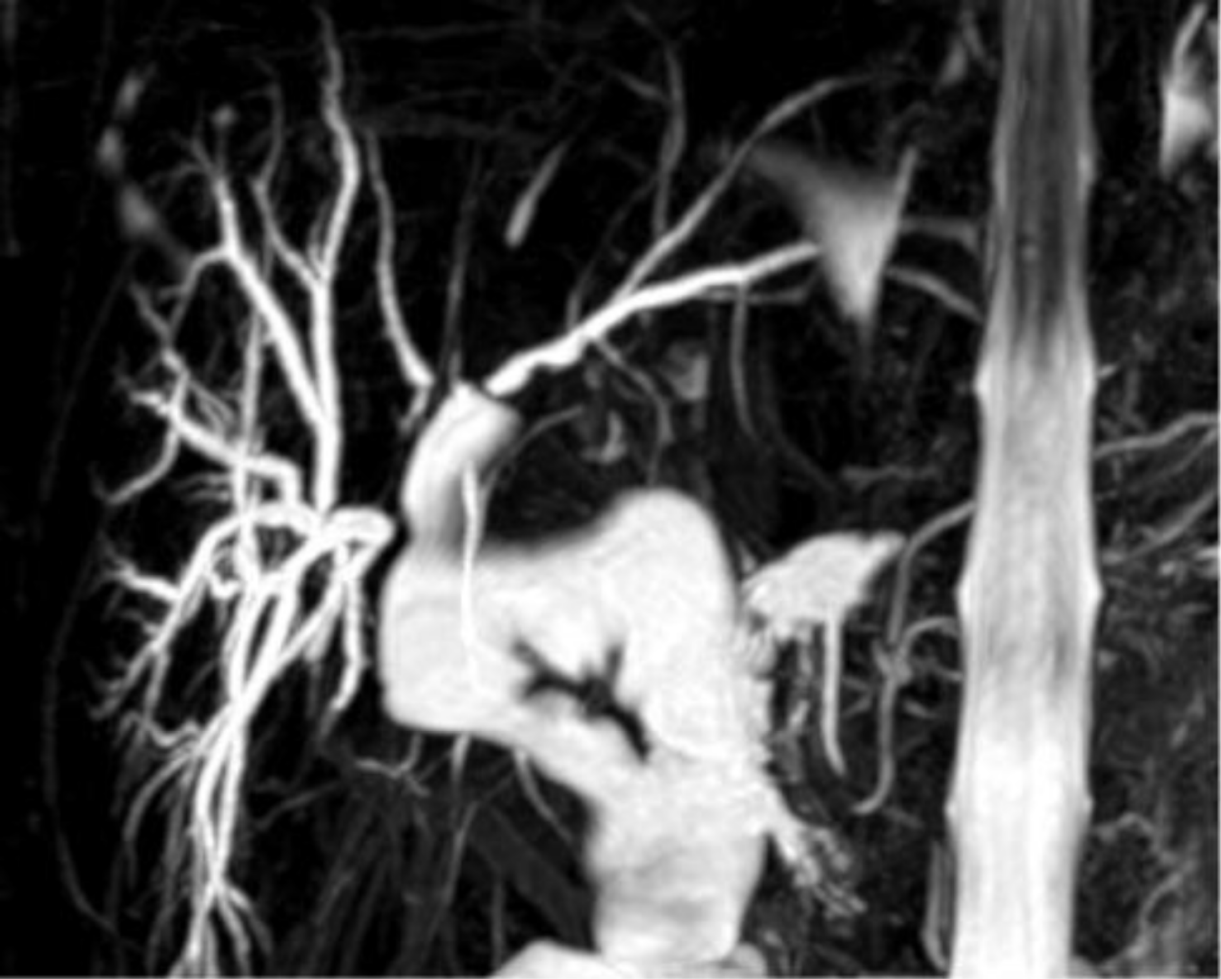
Dilated and excluded bile duct of segment four.

The patient presented to the emergency department with severe cholangitis, requiring hospitalization. Upon admission, cholestasis indices were moderately altered, with bilirubin at 1.8 and GGT at 56. We performed MR cholangiography, which revealed a dilated bile duct in the fourth segment that was excluded from the biliodigestive anastomosis (**Figure 4**).

**Figure 5:**
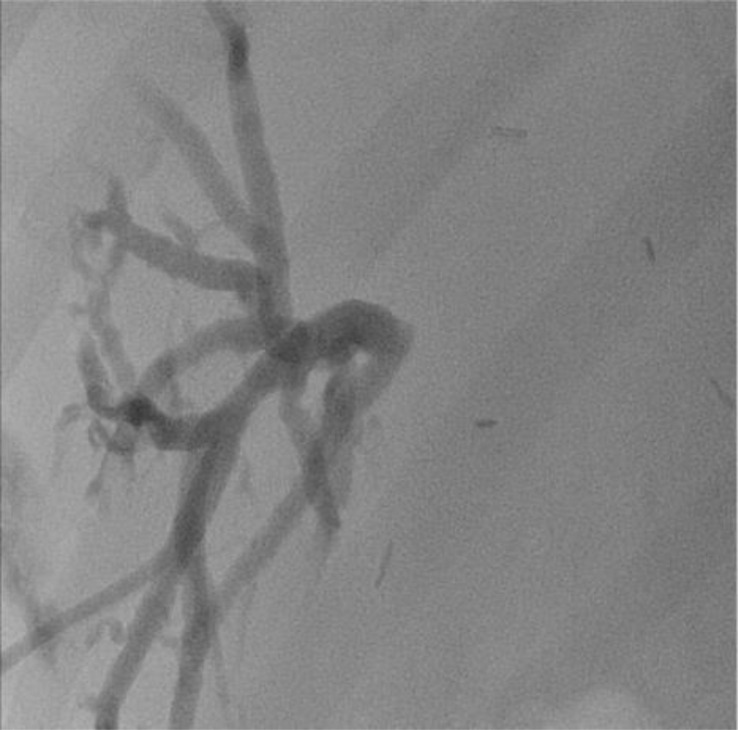
The contrast imaging shows complete exclusion of the dilated bile duct.

Given the clinical presentation and radiological findings, we decided to proceed with percutaneous puncture, aiming to dilate the stricture and restore continuity in the bile ducts. Unfortunately, the procedure was unsuccessful, as the percutaneous cholangiography (**Figure 5**) demonstrated complete exclusion of the bile duct, and it was impossible to establish continuity with the biliodigestive anastomosis or the rest of the biliary system. Upon reviewing the post-procedure radiological images, we also found that the portion of parenchyma associated with the excluded duct was minimal. Thus, the percutaneous puncture proved futile and likely unnecessary.

After injecting contrast medium into the excluded duct, we opted to maintain an external drain, which was extremely difficult to manage. Its removal was complicated and repeatedly delayed due to episodes of cholangitis following attempts to close the drain, and bile leakage at the insertion site. Bacteriological data obtained from cultures of bile collected from the drain allowed us to initiate targeted antibiotic therapy, which enabled us to remove the drain without complications approximately 3 months after the percutaneous puncture. With ongoing prophylactic antibiotic treatment administered in 6-month cycles, the follow-up has been uneventful so far. Notably, the exceedingly limited extent of hepatic parenchyma involved favored a conservative management strategy. This decision reflected the concern that resecting only a few cubic centimeters of parenchyma through laparotomy—following an extensive and technically demanding adhesiolysis—could entail risks disproportionate to the expected benefits. In the event of recurrent cholangitis or radiological deterioration, retransplantation would represent the inevitable subsequent step in this patient’s therapeutic pathway.

#### Case 3

**Figure 6:**
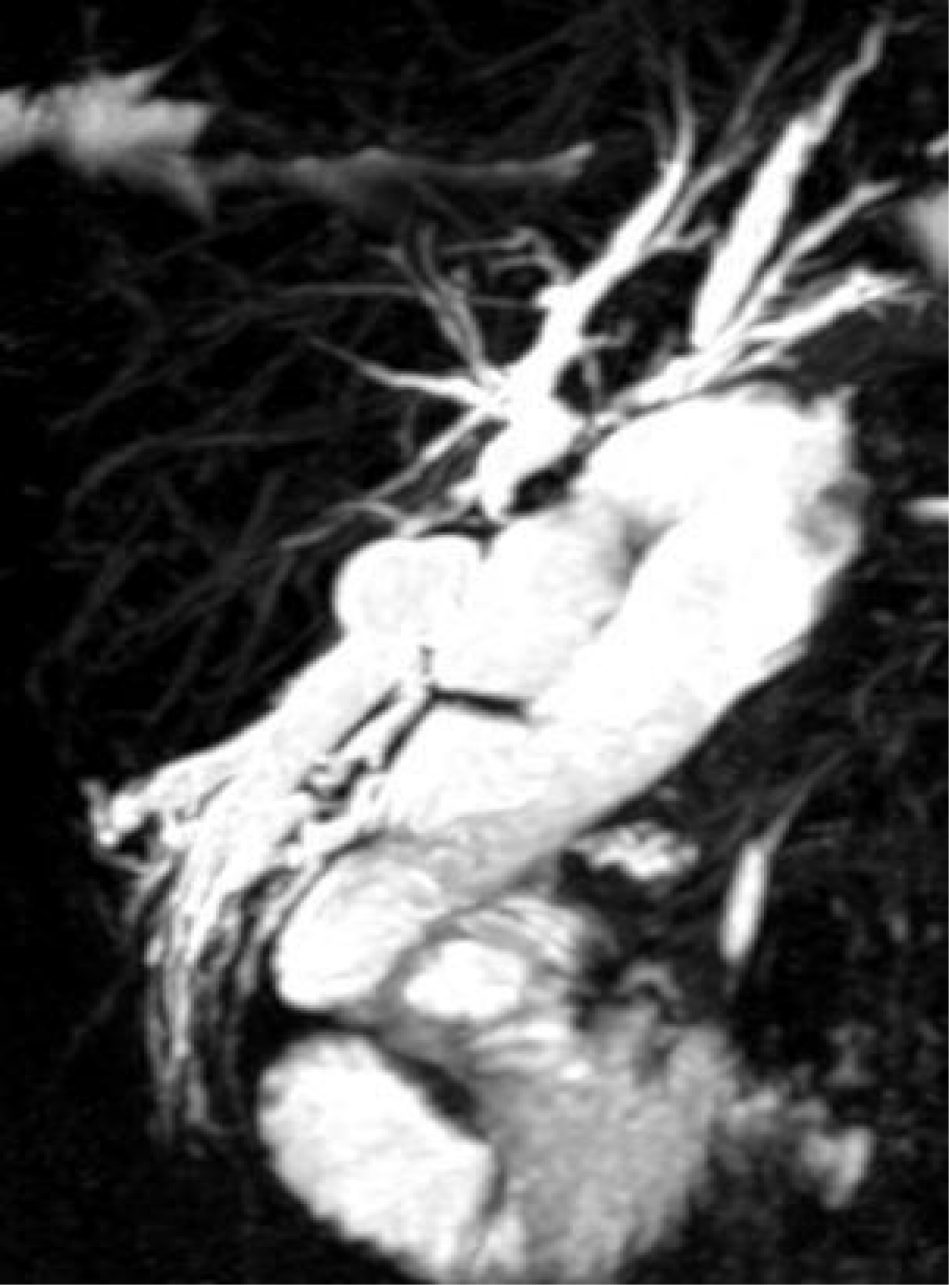
Segmental dilation of the duct for the seventh segment in the presence of a biliodigestive anastomosis.

We managed the third case we present a few weeks after the one just described. In this instance, we faced a stricture of a bile duct in the tributary of the seventh segment in a 23-year-old female patient who had undergone a liver transplant around one year of age due to cholestatic disease. As in the previous case, the initial symptoms presented as recurrent cholangitis. Similarly, we proceeded with MR cholangiography, which confirmed the diagnosis (**Figure 6**).

Following a multidisciplinary discussion with interventional radiologists, in this case we opted for a conservative approach, refraining from invasive procedures. We decided against percutaneous cholangiography as, after a thorough review of the images, we considered the case to be similar to the previous one, with a substantial inability to resolve the clinical situation using this method. In the absence of culture data, we initiated empirical antibiotic therapy in cycles, achieving a positive clinical outcome so far, with approximately 5 months of follow-up without cholangitic episodes. The alternatives we considered were significantly more demanding and complex for the patient, particularly a posterior hepatectomy on the transplanted liver or a potential reassessment with a view toward retransplantation. With a close follow-up plan in place, these options will need to be reconsidered depending on the patient’s clinical progression. In this case as well, the decision to refrain from surgical intervention was primarily driven by concerns regarding the risks associated with subjecting the patient to a lengthy and complex adhesiolysis in preparation for a hepatic resection potentially complicated by intraoperative bleeding and vascular injury. Should symptoms recur, the most appropriate strategy for the center would likely involve the placement of a percutaneous biliary drain, as part of a management pathway oriented toward retransplantation.

#### Case 4

**Figure 7:**
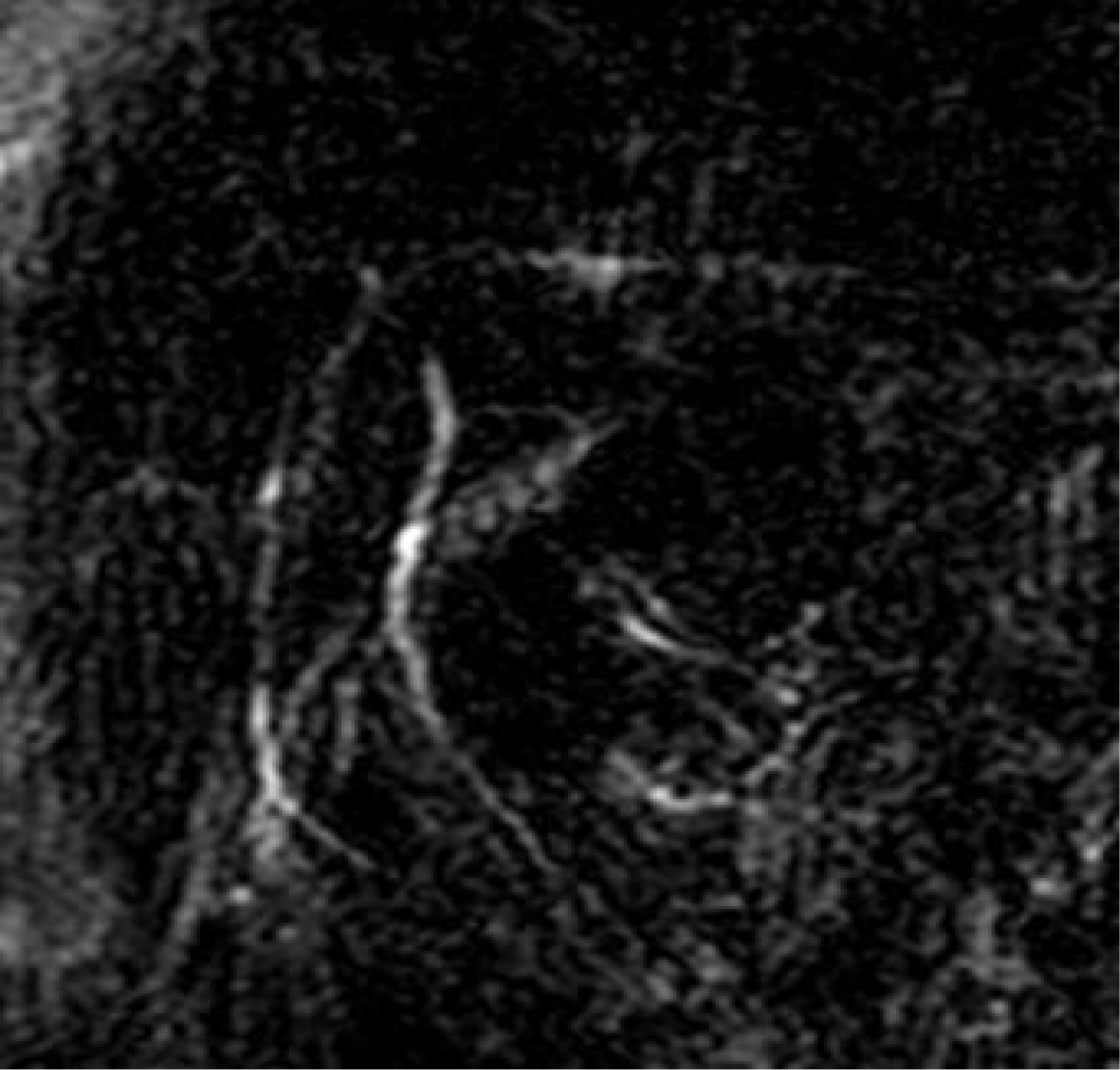
Severe stricture of the biliodigestive anastomosis on MR cholangiography.

**Figure 8:**
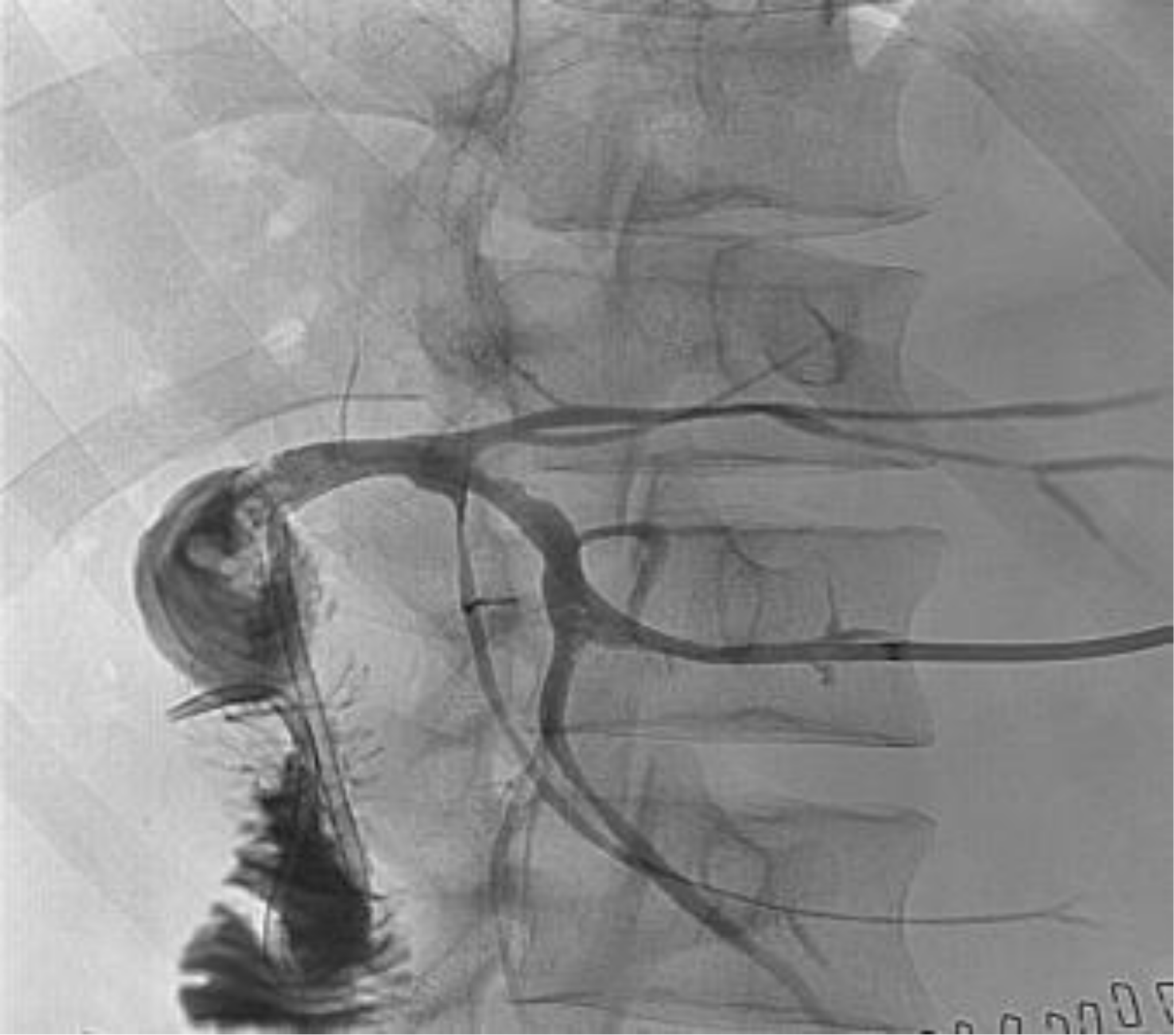
The percutaneous cholangiography demonstrates the restoration of proper bile flow.

The fourth case we report involves a 28-year-old patient who underwent a liver transplant at the age of one for biliary atresia, receiving a left lobe transplant from a living donor (his mother). Despite a nearly regular follow-up, 22 years post-transplant, the patient developed a severe stricture of the biliodigestive anastomosis. As in previous cases, the diagnosis was made following a severe episode of cholangitis via MR cholangiography (**Figure 7**). Despite attempts to resolve the issue percutaneously, the severity and rigidity of the stricture necessitated surgical reintervention, with a reconstruction of the biliodigestive anastomosis. The restoration of proper bile flow after the anastomosis reconstruction was demonstrated by percutaneous cholangiography, performed through a drainage tube placed during the attempt at bilioplasty and maintained in place to protect the future biliodigestive anastomosis (**Figure 8**).

### Other patients with biliary complications

As mentioned, among the 40 patients with over 20 years of post-transplant follow-up, we identified 4 additional patients with a radiological diagnosis (MR cholangiography) of late biliary complications, who were either asymptomatic or presented with minimal symptoms, such as occasional itching responsive to antihistamines. All 4 of these patients experienced at least one episode of cholangitis during their follow-up, but they are currently in good general condition and asymptomatic. The confirmatory diagnosis was thus largely made via MRI performed during periods of clinical well-being, despite elevated GGT levels. Indeed, all of these patients consistently exhibit GGT levels above 400 IU/L, although their bilirubin levels and liver function indices remain within normal ranges.

In these patients, a conservative treatment approach was chosen for various reasons during their individual follow-ups. They are enrolled in a monitoring protocol that includes biannual ultrasound examinations and annual or biennial MR cholangiography, unless otherwise indicated by clinical changes or the onset of symptoms.

**Figure 9:**
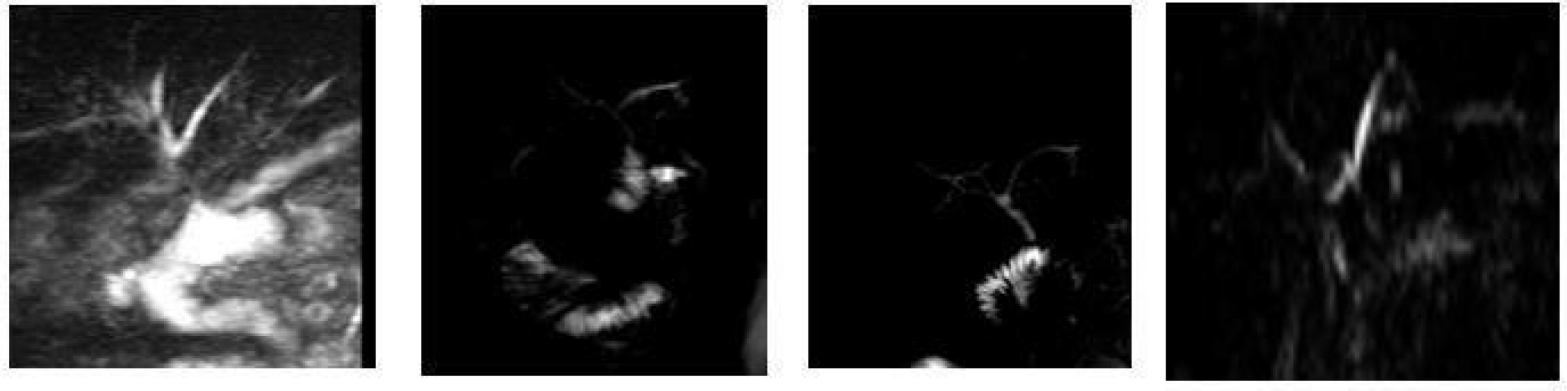
The latest magnetic resonance imaging in asymptomatic patients with elevated GGT levels.

These are the patients for whom interventional options, discussed at a multidisciplinary level at our center will be considered if there are changes in their clinical condition, such as the appearance of symptoms, or significant alterations in radiological findings. The most recent MR cholangiography images of these patients are shown in **Figure 9**.

## Discussion

The management of biliary complications is one of the most frequent challenges faced by transplant surgeons. Biliary complications, which affect 20-30% of transplanted patients according to the major studies in the literature, may be increasing, likely due to the use of extended-criteria donors such as elderly or steatotic donors, as well as the growing use of donors after circulatory death (DCD) across Europe (10, 11).

While the management of early complications, such as anastomotic leaks or strictures of both end-to-end and biliodigestive anastomoses, is part of the standard skillset of clinicians working in transplantation, and is relatively straightforward in most cases, the treatment of late-onset complications, such as anastomotic or intrahepatic biliary strictures, proves to be more complex and nuanced (12). These complications often suggest an underlying ischemic issue that complicates treatment and, in severe cases, may lead to the need for retransplantation (13). As specified in the Materials and Methods section, four patients in our historical cohort also underwent retransplantation for biliary complications before reaching twenty years of follow-up.

A distinct yet emerging subgroup within the wide and diverse population of liver transplant patients comprises those who underwent transplantation in childhood. Today, these patients have a long follow-up history and a substantial future life expectancy (14). In this context, every clinical decision must consider these factors (10).

In our experience, after analyzing a classic case of biliodigestive anastomotic stricture in a pediatric transplant patient who received a left split graft, treated with common but still complex interventional radiology techniques, such as percutaneous bilioplasty, we recognized what became a strategic and management error in the long run. In the second case presented in our results, we describe an extremely challenging experience for our center and, more importantly, for the patient we treated. By inadvertently targeting an excluded bile duct with no possibility of restoring proper bile outflow, we condemned the young patient to three months of external biliary drainage, with no chance of removal or closure. This significantly worsened her quality of life, essentially preventing her from attending school regularly. Minor management complications, such as bile leakage at the drainage insertion site, only compounded this already precarious situation. The sole positive outcome of this drainage placement was obtaining a bile culture, which allowed us to initiate targeted antibiotic therapy that has, so far, produced good results. The patient has remained free of drainage and cholangitic episodes for approximately six months. This case led us to adopt a more cautious and less interventional approach, as demonstrated in the third case, and prompted us to conduct a more thorough review of imaging. We focused not only on biliary anatomy and the local complication but also on the volume of parenchyma involved in the excluded segment, which, in some cases, may atrophy spontaneously, as we observed upon reviewing the MR cholangiograms of both girls in cases 2 and 3. It is clear that the conservative treatment we implemented in both cases, involving prophylactic antibiotic therapy and close surveillance, does not preclude the possibility of future interventions with more aggressive approaches, such as hepatic resections or listing for retransplantation, if the less drastic options are deemed to have failed or become futile. A stepwise approach is, in fact, the one we employed in the patient described in case number four. In cases where a side-to-side anastomosis has been performed between the biliary tract of the graft and that of the recipient, percutaneous or endoscopic interventional options remain the treatment of choice in most centers. Surgical options are generally reserved for cases refractory to these treatments. In case number four, the impossibility of performing percutaneous bilioplasties led us to schedule a surgical intervention to reconstruct a biliodigestive anastomosis. When such an indication arises, it is mandatory to carry out a thorough study of the vascularization of the transplanted liver to confirm adequate arterial supply to the graft and the primary biliary convergence where the new anastomosis will be performed. At our center, in addition to routine Doppler ultrasound surveillance, we systematically perform contrast-enhanced CT imaging prior to any surgical or interventional radiology procedure on the biliary tract, in order to assess the patency of the hepatic artery. In planning the interventional management of biliary complications, some promising options have recently been proposed, which may become part of the management protocols for these patients in the coming years. The Barcelona group (15) recently presented positive results on the use of biodegradable stents in managing anastomotic strictures. Although our experience with this technique is currently limited and with less satisfactory results, the potential contribution these options may offer to the non-surgical management of these patients is undoubtedly worth considering. The aspects we consider most interesting in this study relate to the conservative management of patients under our care with biliary complications (intrahepatic strictures involving the anastomosis in a clinically insignificant manner), identified through MRCP and characterized by elevated GGT levels. In such cases, we deemed it prudent to follow a close monitoring protocol, including biannual ultrasound and blood tests, and annual MRCP in the absence of significant clinical or laboratory changes. This approach partially contrasts with other reports in the literature, which suggest more aggressive interventional approaches (16–18) often driven by concerns that potential clinical deterioration or repeated episodes of cholangitis could worsen biliary damage and progressively affect graft function. However, this must be weighed against the relatively high risk of inducing cholangitic events through percutaneous procedures, which carry the associated risk of septic complications. The group from Bergamo (18) recently described their experience with these patients, confirming that while the risk of post- procedural cholangitic events is relatively high, the clinical impact is often moderate. The colleagues from the Bergamo group also emphasize the importance of obtaining bile cultures via drainage, in order to initiate targeted antibiotic therapy. In our experience, a program of close clinical monitoring can support a more conservative, wait-and-see approach. In more doubtful and complex cases, significant decision- making support may come from pathological data obtained through liver biopsy. Many centers, including ours, continue to follow protocol biopsy programs every five years in pediatric transplant patients. The pathological data are essential in evaluating fibrosis and detecting signs of acute and chronic rejection and can also be directed toward assessing the condition of the bile ducts and detecting signs of cholangitis in the graft (19). In addition to protocol biopsies, in patients with elevated GGT levels and an unclear radiological picture, histological assessment of biliary damage could assist in guiding management towards a more or less interventional approach. In our case described as number 3, for example, a biopsy negative for chronic cholangitic changes in the left lobe parenchyma supported our decision to implement a “watch and wait” protocol, although we are fully aware that this approach requires caution and careful monitoring—particularly with the need to escalate to more invasive treatments in cases of recurrent infections, which may complicate the clinical course and make potential retransplantation more challenging. The role of antibiotic prophylaxis in these patients remains controversial. A recent review and meta-analysis (20) conducted in a different patient setting, specifically children who underwent Kasai procedure for biliary atresia, showed no differences in terms of patient outcomes or in prolonging the life of the native liver without the need for transplantation. Although there is no clear evidence (21) in the long-term transplanted population that prolonged antibiotic prophylaxis improves outcomes, and indeed, infectious disease specialists are rightly concerned about the emergence of antibiotic resistance due to inappropriate antibiotic prescriptions, in selected cases, targeted therapy based on culture results could be considered. In any case, a significant study by the Barcelona group recommends the systematic use of antibiotic prophylaxis in all pediatric patients undergoing biliary interventional procedures. The pathogens most frequently responsible for post-procedural cholangitic conditions identified by the Spanish colleagues were Gram-negative (64%), Gram-positive cocci (28%), and Candida spp. (8%). This information should be carefully considered when contemplating specific antibiotic prophylaxis for this patient population (22, 23).

## Conclusions

The number of pediatric liver transplant recipients with long-term follow-up, often exceeding 20 to 30 years, is increasing. These patients are unique due to their extensive medical histories and their long life expectancy. Therefore, transplant-related complications, especially biliary ones, which are the most common, require careful and tailored management. Traditional interventional approaches have proven effective in addressing most of these complications, reserving more invasive surgical and re-transplantation options for highly selective cases. In our experience, a subset of these patients may benefit from a more conservative approach, provided that strict and attentive follow-up is maintained, even if this option should not delay or replace radical intervention when the latter is feasible and potentially curative.

## Data Availability

The raw data supporting the conclusions of this article will be made available by the authors, without undue reservation.
